# On the horizon: Hedgehog signaling to heal broken bones

**DOI:** 10.1038/s41413-021-00184-8

**Published:** 2022-02-15

**Authors:** Stephanie T. Kuwahara, Shuwan Liu, Andrew Chareunsouk, Maxwell Serowoky, Francesca V. Mariani

**Affiliations:** University of Southern California, Keck School of Medicine, Department of Stem Cell Biology and Regenerative Medicine, Los Angeles, CA 90033 USA

**Keywords:** Bone, Bone quality and biomechanics

## Abstract

Uncovering the molecular pathways that drive skeletal repair has been an ongoing challenge. Initial efforts have relied on in vitro assays to identify the key signaling pathways that drive cartilage and bone differentiation. While these assays can provide some clues, assessing specific pathways in animal models is critical. Furthermore, definitive proof that a pathway is required for skeletal repair is best provided using genetic tests. Stimulating the Hh (Hedgehog) pathway can promote cartilage and bone differentiation in cell culture assays. In addition, the application of HH protein or various pathway agonists in vivo has a positive influence on bone healing. Until recently, however, genetic proof that the Hh pathway is involved in bone repair has been lacking. Here, we consider both in vitro and in vivo studies that examine the role of Hh in repair and discuss some of the challenges inherent in their interpretation. We also identify needed areas of study considering a new appreciation for the role of cartilage during repair, the variety of cell types that may have differing roles in repair, and the recent availability of powerful lineage tracing techniques. We are optimistic that emerging genetic tools will make it possible to precisely define when and in which cells promoting Hh signaling can best promote skeletal repair, and thus, the clinical potential for targeting the Hh pathway can be realized.

## Introduction

**Fig. 1:**
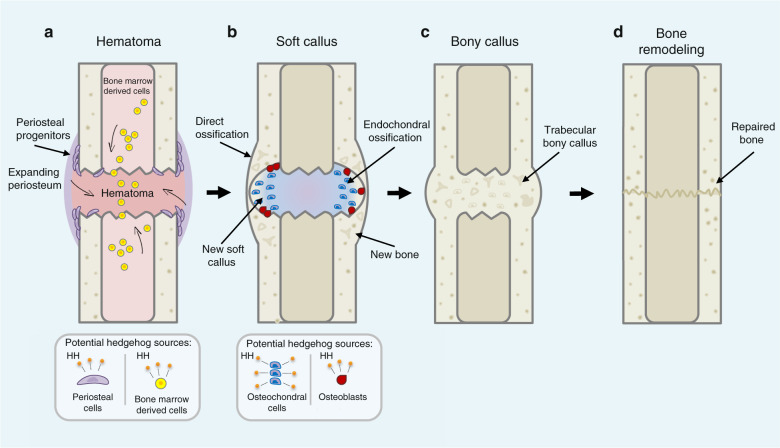
Stages of fracture repair. **a** A hematoma forms as the initial response to fracture injury. During this time, periosteal cells activate (purple), expand in number, and are recruited to the injury site alongside bone marrow-derived cells (yellow). At this stage, both periosteal cells and bone marrow-derived cells are potential sources of a HH (hedgehog) signal (orange). **b** New cartilage matrix is deposited at the fracture sites (blue), giving rise to the early soft callus. Direct ossification occurs concomitantly in the expanded periosteal layer as osteoblasts (red) assemble new bone, and together with the newly formed cartilage, the soft callus forms to bridge the fracture gap and stabilize the injury site. Along with periosteal cells and bone marrow-derived cells, osteochondral cells and osteoblasts could be sources of HH ligands. **c** The hard bony callus is formed as the cartilage matrix is resorbed and calcified through endochondral ossification. New woven bone established by osteoblasts undergoes remodeling and is replaced by trabecular bone, and a secure union between fracture ends is formed. **d** The hard callus is further remodeled, replacing trabecular bone with lamellar cortical bone. At this point, the union of the fractured ends is complete

Although the human skeleton can often repair simple fractures, nonhealing fractures and large-scale critical-sized defects are still major clinical challenges.^1,2^ Typically, after a bone fracture, a hematoma forms, followed shortly by an acute inflammatory phase. Skeletal progenitors become active, move to the injury site, and differentiate (reviewed in ref. ^3^). If repair occurs through endochondral ossification, a soft, unmineralized cartilage callus forms, followed by the establishment of a hard mineralized callus, and finally, the callus undergoes remodeling to form lamellar bone surrounding the bone marrow cavity (see Fig. 1). If the bone repair occurs through intramembranous ossification, skeletal progenitors differentiate directly into bone. While these main events during fracture repair have been well described in the literature, in either scenario, the repair process is complex, involving many cell types and signaling pathways that must coordinate to restore the injured bone. Which signaling pathways are required and how they converge to facilitate bone repair in different contexts are still unclear. Recent studies have begun to examine several signaling pathways during fracture repair, with an emphasis on those pathways that have known roles in bone development (as reviewed in refs. ^4,5^). The Hedgehog (Hh) signaling pathway has a well-known role in bone formation, and recent studies suggest that stimulating this pathway may enhance bone repair. Thus, this review will examine recent efforts to determine the precise role of Hh signaling during bone repair and define its clinical potential.

**Fig. 2:**
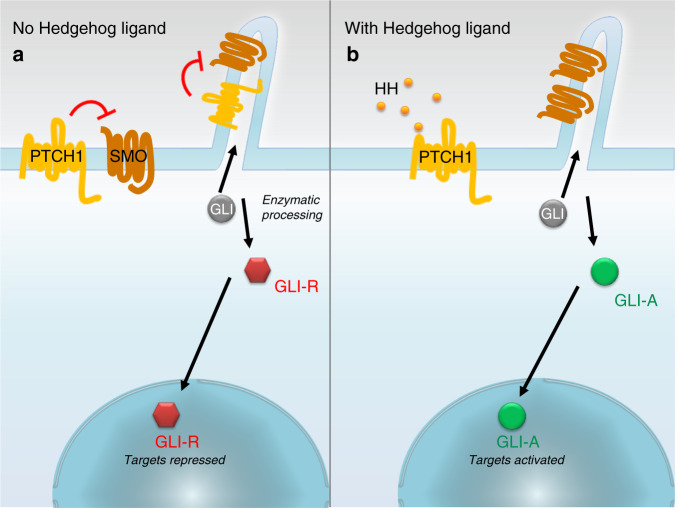
Simplified schematic of Hedgehog signaling. **a** In the absence of HH (hedgehog), the ligand PTCH1 (PATCHED) inhibits SMO (SMOOTHENED) and prevents SMO from translocating to primary cilia. In the absence of SMO, GLI (glioma-associated oncogene) transcription factors are enzymatically processed into their repressor form and act to negatively regulate canonical Hh-controlled genes. **b** In the presence of Hh signaling, the inhibition of SMO by PTCH1 is relieved, allowing SMO to translocate to primary cilia. SMO translocation to primary cilia allows the enzymatic processing of GLI transcription factors into their active form, allowing them to activate Hh-controlled gene expression

**Fig. 3:**
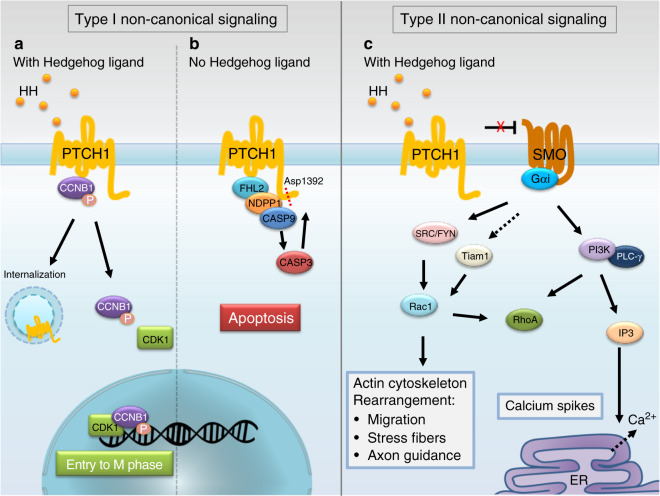
Type I and Type II noncanonical hedgehog signaling. Type I signaling does not require the SMO and GLI transcription factors and is mediated by PTCH1. **a** PTCH1 binds to and sequesters the active, phosphorylated form of cyclin B1 (CCNB1). In the presence of the HH ligand, PTCH1 is internalized, allowing CCNB1 to associate with CDK1 (CYCLIN DEPENDENT KINASE 1) to form the M-phase promoting factor and translocate into the nucleus to initiate entry into M phase. **b** PTCH1 can also regulate cell survival. In the absence of the HH ligand, PTCH1 assembles with a proapoptotic multiprotein complex including FHL2 (also called Dral) and NDPP1 (also called Card8 or Tucan) and contains a dependence-associated receptor C-terminal motif that is cleaved by caspases at a conserved aspartic acid (Asp1392) to expose a proapoptotic domain. Type II signaling requires SMO but transduces HH signals via a nontranscriptional mechanism. For more details, see these reviews.^12,13^ **c** This type of signaling mostly relies on SMO as a G protein-coupled receptor (GPCR) and the involvement of numerous small GTPases, regulating cytoskeletal remodeling, calcium influx, and metabolic reprogramming. These proteins include those in the Src kinase family (e.g. SRC and FYN but perhaps others as well), TIAM1, or PI3K. SMO can stimulate calcium release from the endoplasmic reticulum in spinal neurons through GNAI1 (Gαi)- and PLCG2 (PLC-γ)-catalyzed generation of IP3 and the opening of IP3-dependent calcium channels^106^

Much is known about Hh signaling,^6^ but for the purpose of this review, its role in bone and cartilage will be the focus. In brief, canonical signal transduction in vertebrates involves the expression of one or more of the HH ligands (3 in the family): Sonic hedgehog (SHH), Indian hedgehog (IHH), and Desert hedgehog (DHH), which are expressed at distinct locations and times.^7^ All HH ligands bind to and inhibit the activity of the Patched protein (PTCH1), a highly conserved 12-pass membrane receptor. In the absence of ligand binding, PTCH1 inhibits Smoothened (SMO), a G protein-coupled receptor (GPCR). When the HH ligand binds to PTCH1, this inhibition is relieved, and SMO is free to activate its downstream targets, the GLI transcription factors, which convert from their repressor form to an activator form and drive changes in gene expression (see Fig. 2).^8–10^ In vertebrates, processing of the GLI proteins is concentrated at the primary cilium of the cell (reviewed in detail in ref. ^11^). HH ligands can also activate noncanonical signaling, which does not require the full components of the canonical signaling pathway and can be subdivided into at least two major types based on the subset of components required (reviewed in refs. ^12,13^). Type I signaling does not require the SMO and GLI transcription factors but is dependent on PTCH1 and HH ligands and predominantly regulates proliferation and cell survival. Type II signaling involves HH ligands and PTCH1 but relies on SMO as a GPCR and its interaction with numerous GTPases to regulate cytoskeletal remodeling, calcium influx, and metabolism (Fig. 3). A third type of signaling is independent of HH ligands, PTCH1, and SMO and instead involves both direct and indirect activation of GLI via a wide variety of players ranging from those in the MAPK, PKC, and PI3K/AKT pathways to oncogenes/tumor suppressors and epigenetic factors (reviewed in ref. ^14^). A few emerging studies consider the activity of noncanonical signaling; however, most research has focused on the canonical signaling pathway.

During early skeletal development, Hh signaling is known to have a wide variety of biological roles, including promoting proliferation, cell survival, and differentiation.^15^ In addition, it has well-characterized roles at the growth plate and later during adult bone turnover.^16–18^ Although Hh signaling has been investigated in the context of fracture repair, which cells require Hh signaling and when is not clear. Many studies have primarily relied on assessing the final outcome of the repair process—bone formation. While this method may be appropriate in the context of repair via intramembranous ossification, the focus on late-stage bone formation ignores a potentially key step during endochondral ossification, the generation of the intermediate cartilage callus that prefigures bone. Inattention to this early stage during analysis and, as described below, conflicting results from in vitro and in vivo studies have made it difficult to determine the specific role of Hh signaling during the repair process. Additionally, largely due to the lack of precise tools available, determining the specific requirement for Hh signaling in progenitor subpopulations has been challenging. The purpose of this review is to summarize and discuss the current body of literature investigating the role of Hh signaling during both intramembranous and endochondral bone repair, as well as highlight key questions that remain unanswered. Additionally, we emphasize the importance of assessing the generation of the cartilage callus intermediate during endochondral repair. While most experiments discussed here were performed using mouse models, we also highlight studies using other models, including zebrafish, rabbits, and human-derived cells. A summary of the key studies is included in Tables 1 and 2.

**Table 1:** Summary of studies in which skeletal repair has been assayed with enhanced Hh signaling

Reference	Injury type	Hh GOF	Assay	Results	Conclusions
^38^	Rabbit 8-mm cranial defect	*Shh*-transduced gingival fibroblasts Periosteal-derived cells MSCs Fat-derived cells	Gross anatomy Radiography Histology	• Substantial bone when compared to controls at 6 and 12 weeks	• SHH enhances osteogenic differentiation
^42^	Mouse 4-mm cranial defect	Hh agonist (SAG) *Osteoinductive scaffold*	MicroCT Histology	• Increased bone when compared to controls at 4 and 8 weeks	• SAG suppresses adipogenic differentiation while actively promoting osteogenic differentiation
^29^	Rat 2.2 mm cylinder-shaped femur shaft defect	Hh agonist (SAG) *Tetrapod* Ca^2+^ PO_4_^3−^ *granules*	Histology MicroCT	• Higher BV/TM, bone mineral content, and bone density compared to controls	• SAG initially and transiently specifies host cells into the osteoblastic lineage
^28^	Rabbit tibial defect	IHH-expressing MSCs *HA scaffold*	Histology MicroCT Gross Anatomy	• New bone and cartilage, well-arranged capillary network at 4 weeks • Complete healing at 12 weeks	• IHH enhances chondrogenesis, osteogenesis, and angiogenesis but not cell survival or proliferation
^33^	Mouse femoral 4-mm segmental bone allograft	SHH-N-expressing PDMPCs *Collagen scaffold*	X-ray MicroCT Histomorphometry	• More donor cells present, increased CD31^+^ microvessels at 4 weeks • Complete bridging at 6 weeks with less fibrotic tissue, larger and more mature vessels	• SHH enhances osteogenic differentiation, donor cell survival, and neovascularization
^40^	Mouse femur fracture	Hh agonist (Hh-Ag1.5)	MicroCT Strength testing Histology	• Larger callus and bone volume, mechanical strength, and vascularity compared to controls	• Hh-Ag1.5 administration improves the osseous and vascular healing response

**Table 2:** Summary of studies in which skeletal repair has been assayed with reduced Hh signaling

Reference	Injury type	Hh LOF	Assay	Results	Conclusions
^34^	Mouse 4-mm femoral autograft	*Rosa-CreER;Smo*^*f/f*^	MicroCT Histology	• Reduced bone and callus formation • More undifferentiated cells and fibrotic tissue • Reduced proliferation	• Hh signaling drives expansion of the early repair callus
^30^	Mouse tibial fracture	*Esr1-Cre;Smo*^*f/f*^	Radiography Histology Histomorphometry	• Smaller callus, 28 days post-fracture	• Hh signaling modulates repair quality by enhancing osteoblast differentiation but is not required
		*Col2-Cre;Smo*^*f/f*^		• No change, 28 days post-fracture	• Hh signaling is dispensable for chondrocytes
		*Col1-Cre;Smo*^*f/f*^		• Smaller callus, decreased bone tissue, 28 days post-fracture	• Hh signaling promotes osteoblast activity and matrix production
^43^	Mouse fibular fracture	*Col2a1-Cre;Ihh*^*f/f*^	X-ray MicroCT Immunohistochemistry	• Reduced cartilage callus but no change in bone formation or overall fracture repair	• Hh signaling affects cartilage but is not required for fracture repair
^60^	Zebrafish mandibular resection	*Ihha* mutant	Histology Alizarin red/Alcian blue RNA-ISH MicroCT	• Reduced cartilage callus and bone formation, poor bone quality	• Hh signaling is required for cartilage induction during regeneration
^44^	Mouse Rib resection	*Sox9-CreER;Smo*^*f/f*^	Histology Alizarin red/Alcian blue RNA-ISH	• Reduced cartilage callus and bone formation	• Hh signaling is required for cartilage induction during regeneration

### Hh and its role in osteogenesis during development

During pre- and postnatal bone growth, regardless of location and developmental mechanism (intramembranous vs. endochondral), Hh signaling has been shown to be required for osteoblast differentiation. In the cranium, both the SHH and IHH ligands are expressed at the osteogenic front of the expanding calvarial bones.^19,20^ Mutational analysis of *Ihh* showed that it is required for osteogenesis, and a careful analysis demonstrated that IHH is not required for progenitor proliferation but for their differentiation into mature osteocytes.^20^ Another study identified cells expressing the Hh pathway transcription factor *Gli1* in the suture mesenchyme and, after 2 months of lineage tracing (using *Gli1-CreER* mice), detected these cells in the periosteum, dura, and parts of the calvarial bones. Ablation of these cells at 1 month of age caused craniosynostosis and osteoporosis and arrested skull growth. Conditional inactivation of Hh signaling using *Gli1-CreSmo*^*fl/fl*^ animals, however, only revealed an osteoporotic defect 8 months after tamoxifen induction and no obvious changes in progenitor proliferation.^21^ Interestingly, intramembranous bone formation in the prechordal cranium does not appear to require the HH ligand. However, mouse genetic knockout experiments have shown that the Hh pathway is regulated by noncanonical Gαs signaling to control osteoblast commitment and maturation rather than their proliferation.^22^ Taken together, these results suggest that Hh pathway activation in the skull, regardless of the location and dependency on ligands, is more important for osteogenic differentiation than establishing progenitor pools.

In the appendicular skeleton, which undergoes endochondral ossification, a bone collar first forms when perichondrial cells undergo osteoblast differentiation. IHH released by neighboring cartilage cells is directly required for this process, as both eliminating *Ihh* from cartilage cells (using *Col2-Cre*) and inactivating *Smo* from both the cartilage template and the perichondrial cells (using *Col2a1-Cre3* and *Col2a1-Cre10*) result in a failure to form bone collar osteocytes.^23–25^ As the growth plate forms at either end of the bones, IHH is produced by differentiating chondrocytes, and if IHH production is blocked (using *Col2-Cre*), osteoblast differentiation and growth plate organization are disrupted.^26^ *Gli1*-expressing cells are abundant in the growth plate and sparse in the periosteum of long bones and presumably represent the cells responding to an Hh signal. Elimination of *Smo* in these cells using *Gli1-CreER;Smo*^*fl/fl*^ mice resulted in decreased bone formation and fewer *Osx*-expressing cells in the trabecular region just below the growth plate, likely due to decreased proliferation, as shown by EdU incorporation analysis.^27^ Postnatally, osteoblasts also produce IHH, which can promote osteoblast proliferation, survival, and differentiation. However, interestingly, if sustained, Hh signaling leads to osteopenia caused by a secondary effect on osteoclasts, bone resorptive cells.^17^ Thus, depending on the context (skull vs. appendicular skeleton) and duration of signaling, Hh signaling can either directly or indirectly influence osteoblast proliferation or differentiation or both.

### Osteogenesis in vitro

The osteogenic inductive capacity of Hh signaling has been demonstrated in vitro with various cell types and conditions. For example, overexpression of IHH in bone marrow MSCs and C3H10T1/2 cells induced an increase in markers of osteogenic differentiation, such as ALP staining and BGLAP (osteocalcin) expression.^28^ Treatment of C3H10T1/2 cells with the Hh agonist (SAG) similarly resulted in the activation of the osteogenic differentiation program.^29^

Stimulating Hh signaling can not only enhance osteogenic differentiation but also promote osteoblast activity, as shown by matrix production. When bone marrow stromal cells carrying a constitutively active SMO receptor (from *Col1-Cre;Smo*^*Stab*^ mice) were cultured under osteogenic conditions, increased ALP staining was observed compared to that of the controls, as well as increased von Kossa staining and *Col1* expression, both indicating an increase in bone matrix production.^30^

The periosteum contains poorly defined progenitor populations that contribute to both bone homeostasis and bone repair.^31,32^ For determination of whether stimulating Hh signaling induces osteogenic differentiation specifically in periosteal progenitors, periosteal-derived mesenchymal progenitor cells (PDMPCs)^33^ and periosteum callus-derived mesenchymal stem cells (PCDSCs) were isolated from healing segmental bone graft injuries, transduced to overexpress the N-terminal SHH peptide (SHH-N, which contains all the signaling functions of the protein), and placed in osteogenic media. Similar to the results using bone marrow-derived MSCs, these cells showed increased expression levels of osteogenic markers such as *Alp* and *Bglap* compared to untransduced controls.^33,34^ Likewise, treating PCDSCs with the Hh agonist purmorphamine strongly enhanced osteogenic differentiation and mineralization compared to those cultured in only osteogenic media.^34^

While the above research supports the sufficiency of Hh signaling in promoting an osteogenic program, other in vitro experiments have also been used to assess the necessity of Hh signaling during osteogenic differentiation. Isolated bone marrow stromal cells with a compromised ability to respond to an Hh signal (from *Col1(2.3* *kb)-Cre;Smo*^*fl/fl*^ mice) were cultured under standard osteogenic differentiation conditions. Decreased ALP and von Kossa staining compared to that of the controls was observed, indicating decreased osteoblast activity and matrix production.^30^ Similarly, PCDSCs were isolated from the *Rosa-CreER;Smo*^*fl/fl*^ mice (80% reduction of *Smo* mRNA expression compared to that of the controls), were cultured in osteogenic differentiation media, and displayed decreased osteogenic potential. Similar results were observed when the control PCDSCs were cultured with the Hh antagonist cyclopamine.^34^ More recently, the SMO antagonist BMS-833923 was shown to inhibit osteoblastic differentiation of human MSCs.^35^ Thus, these results provide strong evidence that the SMO-dependent, canonical Hh pathway is required for the differentiation of both bone marrow-derived and periosteal cells into osteoblasts in vitro.

Emerging evidence in vitro has also suggested the involvement of noncanonical Hh signaling in osteogenesis. For example, culturing MT3T3-E1 preosteoblasts on Matrigel under osteogenic conditions induced a morphological transition, leading to the formation of osteocyte-like dendrites.^36^ Activation of canonical Hh signaling was observed, as demonstrated by *Gli1* and *Ptch1* expression. However, kinases such as SFK, p38 MAPK and p42/p44 MAPK, which have been implicated in noncanonical Hh signaling, were found to be activated by kinome profiling, suggesting the possible involvement of noncanonical Hh signaling during dendrite formation. Although the SMO inhibitor cyclopamine arrested the outgrowth of dendrites, it remains unclear whether disruption of canonical or noncanonical Hh signaling accounts for this result.^36^ Another study investigated the requirement of an intraflagellar transport protein found in cilia (IFT80) in osteoblast differentiation using primary calvarial osteoblast progenitor cells.^37^ Loss of *IFT80* inhibited ciliogenesis and stress fiber formation, eventually resulting in impaired osteogenic differentiation. Interestingly, while the application of SHH induced the activation of both canonical and noncanonical Hh signaling in these progenitors, noncanonical Hh signaling was favored in *IFT80*-deficient progenitors via the SMO-Gαi-RhoA-MLC2/Cofilin-stress fiber axis (see Fig. 3). Inhibition of this axis promoted ciliogenesis and restored canonical Hh signaling. These findings demonstrate that at least during osteoblast differentiation in vitro, IFT80 is essential for the balance between canonical and noncanonical Hh signaling. It remains unclear, however, whether IFT80 promotes osteoblast differentiation directly via noncanonical signaling or indirectly by tipping the balance toward canonical Hh signaling. Clearly, more research is required to elucidate the role of noncanonical Hh signaling in bone formation and the possible reciprocal regulation between canonical and noncanonical pathways.

### Osteogenesis during repair

Although in vitro data are promising indicators that HH ligands act as osteogenic inducers, in vivo studies are necessary to determine which steps are compromised when repair fails and to define which step in repair should be targeted for clinical treatment. A variety of bone repair models have been used to investigate the osteogenic potential of Hh signaling. Although the studies discussed below do not identify a precise mechanism, they do indicate that stimulating the Hh pathway has a positive effect on bone repair by promoting osteoblast matrix production and osteogenic differentiation.

Various methods have been utilized to assess the impact of Hh signaling during bone repair. A common strategy is to implant cells overexpressing a HH ligand into bone injuries of different types. Although these studies have shown that exogenous HH can lead to increased bone formation, which cells respond and how new bone is generated remains unclear.^28,33,38,39^ For instance, in a critical-sized rabbit cranial injury model, implanted cells overexpressing SHH-N resulted in increased bone formation.^38^ Although histological and radiographic analysis clearly showed bone production, whether increased bone formation was due to an increase in osteoblast proliferation, matrix production, survival, or differentiation was not determined. Similarly, in the appendicular skeleton, implanted scaffolds loaded with MSCs expressing IHH were placed in a hole defect in a rabbit tibia.^28^ Although CT imaging and histology indicated enhanced bone production, it is not clear whether IHH stimulated MSCs to differentiate into osteoblasts or whether the IHH produced by MSCs enhanced the proliferation, differentiation, or survival of other cells involved in repair. One study attempted to address the cellular origin by implanting SHH-N-expressing PDMPCs into a murine segmental femoral injury model. Researchers identified more SHH-N-expressing PDMPCs incorporated into a devitalized allograft, including some that had differentiated into osteoblasts, compared to untransduced control cells 2 weeks after implantation.^33^ Together, these studies collectively demonstrate that exogenous HH can lead to increased bone formation, but the precise details that govern this process remain unclear.

In contrast to implanted cell strategies, the administration of Hh agonists can stimulate bone production with better control of dose and timing.^29,40,41^ Scaffolds loaded with the agonist SAG and placed in a murine calvarial defect increased bone formation compared to scaffolds alone.^42^ Similarly, in a study of femur fractures in aged mice (18 months) that typically healed poorly via endochondral ossification, daily systemic administration of the agonist Hh-Ag1.5 resulted in earlier bone callus bridging, increased callus and bone volume, and greater strength, as indicated by mechanical testing, compared to those of the untreated controls.^40^ Likewise, when modeling femur defects in rats, short-term release of SAG from implanted calcium phosphate granules increased bone formation compared to that of the controls.^29^

Thus, given the results of both in vitro and in vivo studies, there is consistent evidence for the capacity of Hh signaling to promote bone formation, offering a promising avenue for future treatments of nonhealing bone injuries with Hh agonists. However, to determine when the administration of an Hh agonist would be most effective (e.g., on mesenchymal precursors prior to osteogenesis, on osteoprogenitors during matrix production, or on osteoblasts to promote maturation during bone remodeling), more precise studies are needed.

Since mice null for *Shh* or *Ihh* die in early gestation, inducible Cre-loxP tools can be used to allow temporal gene removal in adults. Notably, Cre lines have different recombination efficiencies and may not excise their target gene in all cells. In addition, Cre lines can sometimes cause recombination in unexpected cell populations, especially after injury. However, these approaches can help determine when specific cell populations release Hh ligands and when specific target cells respond. For example, *Ihh* was ablated from most *Col2a1*-expressing chondrocytes (*Col2a1-CreERT2;Ihh*^*fl/fl*^ mice) throughout repair of a fibular fracture. If fracture repair is analogous to bone development, the release of IHH from chondrocytes in the cartilage callus might be expected to influence subsequent osteogenesis. However, neither the size of the bone callus nor the timing of bridging was affected in the *Col2a1-CreERT2;Ihh*^*fl/fl*^ mice, although a decreased cartilage callus was observed.^43^ One possibility is that cartilage may not be the primary source of *Ihh* during repair. However, similar results were observed after daily treatments with cyclopamine, an antagonist of SMO, the necessary coreceptor for Hh signaling, which should broadly block all cells from responding to Hh ligands. Together, these data suggest that bone production does not strictly require Hh signaling. However, a caveat to both of these experiments is that low levels of residual Hh signaling (due to incomplete Cre excision, expression of other Hh ligands, or insufficient cyclopamine inhibition) could be sufficient to drive osteogenesis.

As an alternative to modulating the levels of ligands, researchers have genetically targeted the *Smo* gene in bone-producing cell populations during repair. Using an inducible ubiquitous Cre (*Rosa-CreER*), *Smo* was removed during murine femoral autograft surgery.^34^ In contrast to the previously described results, deleting *Smo* led to a decrease in total bone callus formation. However, because Cre in this system is ubiquitous, it is still unclear when and in which cells *Smo* is required, as the cells that make bone were not the only cells targeted. In addition, interpretation of the result is challenging, as increased numbers of cells with an undifferentiated morphology were observed and decreased proliferation was detected in the callus periphery (periosteal side of the repair callus), indicating that Hh could play a role in other lineages and/or at the earlier stages prior to bone formation. Another study deleted *Smo* using inducible ubiquitous *Esr1-CreER* during tibial fracture repair and similarly uncovered mild reductions in total bone callus, although the difference was not statistically significant.

An early role for Hh signaling during repair is supported by RNA in situ hybridization studies showing the expression of *Ihh* in early prehypertrophic chondrocytes of the femur repair callus with HH-responsive cells observed using *Ptch1-LacZ* reporter mice, as early as 3 and 7 days post-injury, in a broad array of cell types, including periosteal cells, chondroprogenitors, chondrocytes, vascular progenitors, mesenchymal cells, and osteoblastic cells forming the new bone.^34^ In addition, *Shh*, a ligand not typically associated with postnatal bone, was observed to be expressed 2 days after rib fracture and 5 days after tibia fracture or rib resection.^33,44–46^ However, which cells express *Shh* is not clear. In an attempt to specifically target *Smo* in bone-producing cells, an osteoblast-specific Cre (*Col1(2.3)-Cre*) was used.^30^ For reasons that are not clear but that may involve more efficient Cre excision, a more dramatic decrease in total bone callus was observed compared to that using a ubiquitous Cre, although bone still did form and bridging was ultimately complete. As the efficiency of *Smo* gene ablation was not assessed, it remains unclear whether Hh signaling is strictly required in osteoblasts/osteocytes, nor is it clear if proliferation or differentiation is the crucial step affected. Nonetheless, these results demonstrate a requirement for Hh signaling during bone repair in vivo. RT-PCR for *Ptch1* and *Gli1* expression, both of which are direct read-outs of Hh signaling, revealed peak expression at 14 days post-fracture, suggesting an important role for Hh during the bone-producing phase of repair. Furthermore, when a constitutively active version of the *Smo* receptor was ectopically expressed throughout repair using either the ubiquitous *Esr1-CreER* or the osteoblast-specific *Col1(2.3)-Cre*, an increase in total bone callus volume was apparent 28 days post-fracture compared to controls.^30^ Thus, although Hh signaling may not be strictly required in osteoblast lineages, stimulating the Hh pathway within *Col1a1*-expressing cells during repair appears to have a beneficial outcome.

### Hh and role in chondrogenesis

While Hh signaling may promote bone repair by regulating osteoprogenitor and osteoblast behavior, Hh signaling may also be critical within the cells that participate in earlier phases of repair, such as the mesenchymal progenitors that first migrate to the injured bone or their differentiation into the cartilage intermediate. Changes in bone formation could then be a secondary consequence of these earlier alterations, especially if the cartilage intermediate is an important prerequisite for bone repair.

Since Hh signaling is required for chondrocyte proliferation in the growth plate, as discussed above and reviewed previously,^47,48^ as well as chondrocyte development during the formation of the skull base and postchordal neurocranium,^49,50^ it may play a similar role in building the cartilage intermediate during repair. In the growth plate, the IHH ligand is produced by prehypertrophic and hypertrophic chondrocytes. Surrounding cells, including chondroprogenitors and overlying perichondrial cells, likely receive the signal. In response, perichondrial cells produce parathyroid hormone-like peptide (PTHLH, also called PTHrP).^51,52^ PTHLH, received by proliferating chondrocytes, promotes proliferation while suppressing their maturation into hypertrophy. As chondrocytes continue to proliferate, it is proposed that their movement farther from the source of PTHLH allows them to differentiate. This negative feedback loop is proposed to allow tight control of chondrocyte proliferation and differentiation.^53,54^ Furthermore, by combining mutants for *Ihh* and *Pthlh* in mice, researchers uncovered an additional PTHLH-independent role for IHH in inducing chondrocyte proliferation, maturation, and the transition of round chondrocytes into a columnar organization.^55–57^

Although most studies have focused on the osteogenic phase of repair, some studies have also investigated the cartilage callus. Recent studies have suggested that improving cartilage callus formation leads to improved bone repair overall.^44,58–61^ Because many bone injuries, especially large injuries due to trauma, result in hypoxic environments, cartilage offers unique reparative advantages due to its low vascular requirements.^62^ Chondrocytes also release VEGF, MMPs, and other growth factors that are beneficial to the repair process.^58,59,63^ Beyond releasing these paracrine effectors, chondrocytes have also been shown to undergo transdifferentiation and/or maturation into osteoblasts and therefore may directly contribute to new bone formation.^44,60,64–66^ Altogether, these studies highlight the potential critical role of the cartilage intermediate during repair and justify further investigations into the role of Hh signaling in the cartilage callus. Furthermore, new therapeutic approaches that modulate the cartilage intermediate phase by influencing growth factor pathways such as Hh, may improve the outcome and speed of bone healing.

### Role of Hh signaling in chondrogenesis in vitro

In vitro studies indicate that activating Hh signaling enhances chondrogenesis, while inhibiting this pathway decreases chondrogenic potential and differentiation. For example, PCDSCs overexpressing SHH-N and placed in micromass culture with BMP-2 demonstrated enhanced chondrogenesis.^34^ Similarly, bone marrow-derived MSCs from rabbit femurs and tibias, C3H10T1/2, and ATDC5 cells that overexpress *Ihh* displayed increased Alcian blue staining compared to controls cultured in chondrogenic conditions.^28,67^ Furthermore, lentiviral-mediated overexpression of *Ihh* and/or *Shh* in rabbit BMSCs substantially enhanced chondrogenic gene expression in a microgravity rotary cell culture system.^68,69^ Conversely, limb mesenchymal cells cultured under micromass culture conditions and treated with HhAntag (Hh inhibitor) exhibited fewer Alcian blue-positive nodules and decreased levels of both early and late cartilage marker genes compared to controls.^70^ In vitro experiments have also shown a role for Hh signaling in promoting the later stages of chondrocyte maturation and calcification. For example, *Ihh*-overexpressing ATDC5 cells cultured with or without a cyclopamine inhibitor showed decreased *Col10a1* expression, an indicator of more mature hypertrophic chondrocytes, as well as decreased mineralization compared to controls.^67^ Together, these experiments indicate that Hh signaling can induce both chondrocyte differentiation and maturation in vitro. However, in vivo experiments are still necessary to determine whether Hh signaling impacts any aspect of cartilage formation or maturation in the context of bone repair.

### Chondrogenesis during repair

While most in vivo studies investigating Hh signaling during repair have focused on bone formation, a few studies have investigated the role of Hh signaling during cartilage callus formation. However, these studies appear conflicting, with results ranging from no role for Hh signaling in chondrogenesis to roles for Hh signaling in either chondrocyte proliferation or chondrogenic differentiation. Some of these seemingly conflicting results may be due to each study employing a distinct injury model. For example, in a report that used fracture assays, reduced Hh signaling was associated with a reduced cartilage callus, but the impact was minor, and bone healing was unaffected. In contrast, the role of Hh may be more significant in the context of a large-scale injury where a bridging cartilage callus is critical. Resolving these differences (discussed in more detail below) will be important for determining the specific role of Hh signaling during bone repair.

The application of Hh pathway modifying agents has had mixed results with regard to chondrocytes during bone repair. In one report, mice with femur fractures were given an Hh agonist (Hh-Ag1.5) orally every day from fracture until analysis. No changes in cartilage formation were observed, indicating that promoting Hh signaling, at least using Hh-Ag1.5, does not affect chondrogenesis.^40^ However, it remains possible that the old age of the mice (18 months) was responsible for the lack of response. In contrast, using MSCs engineered to secrete IHH during repair promoted cartilage formation and complete healing compared to those of defects receiving unmodified MSCs.^28^

In a study where *Smo* was removed using the ubiquitous *Esr1-CreER*, 1 week after tibia fracture when the cartilage callus was typically present, similar amounts of cartilage were observed in both mutant and control mice, indicating that Hh signaling is not required during this stage of cartilage callus formation in a fracture. Similarly, specific deletion of *Smo* in cartilage cells using *Col2‐rtTA‐Cre* did not impact bone callus size or bridging compared to that of controls at 28 days post-fracture.^30^ However, since the analysis in this experiment was carried out at 28 days, it is not known whether the cartilage callus, typically evident at ~1 week, was affected. Furthermore, the observation that bone formation was not affected does not necessarily indicate that a normal cartilage callus formed, since fractures can heal without a cartilage intermediate.^71^

As discussed above, tibial fractures in *Col2a1-CreER;Ihh*^*fl/fl*^ mice where the Ihh ligand produced by chondrocytes is eliminated ultimately healed, and a decreased cartilage callus was demonstrated by safranin O staining and reduced *Col2a1* expression.^43^ While these results support a role for *Ihh* in cartilage callus formation, proliferation assays were not performed, so it was unclear whether in this context, decreased proliferation or failed differentiation was the cause of the reduced cartilage callus. Support for a role of Hh signaling in promoting chondrocyte proliferation can be found in experiments in which *Smo* was deleted with *Rosa-CreER* during bone autograft surgery. Here, reduced proliferation in chondroprogenitors and chondrocytes was observed compared to that in Cre-negative mice.^34^ Although the final volume of cartilage was not quantified in this study, the results suggest that Hh signaling promotes cartilage cell proliferation during repair, as it does at the growth plate.^57^ Ultimately decreased total bone formation was also observed in these animals; however, whether this defect is primarily due to the reduced cartilage callus or a later defect in bone formation remains unclear.

In contrast to fractures, large-scale injuries involve substantial skeletal tissue loss. Thus, the capacity to form a bridging cartilage callus may be particularly important. For example, in a zebrafish model of large-scale repair of the lower jaw, deletion of *ihha* uncovered a dramatic requirement for Hh signaling in establishing a large bridging cartilage callus. Interestingly, the callus failed to form with no significant decrease in proliferation. Reduced bone formation was also observed, although it was not possible to determine if this was due to a role for *ihha* during osteogenesis, a secondary effect of reduced cartilage or both.^60^ By employing a mouse rib resection model that also repairs via a large bridging cartilage callus, our group observed similar results to the zebrafish model. We found that *Smo* is required in callus cells for chondrogenic differentiation. Furthermore, by removing *Smo* from a subpopulation of periosteal progenitors using *Sox9-CreER;Smo*^*fl/fl*^ mice, we observed failed cartilage callus formation, decreased bone formation, and ultimately failed bridging (nonunion). In addition, although cells in the mutant context could fill the callus and coexpress *Sox9* and *Runx2*, they never differentiated into mature callus cells, suggesting that Hh signaling is required to promote chondrogenic differentiation early in the repair process.^44^ These results contrast with fracture studies, where although the callus size was decreased, the fractures ultimately repaired completely.^34,43^ Thus, during large-scale bone repair, Hh signaling may have a conserved role across vertebrate species in promoting the differentiation of a bridging cartilage callus that prefigures the reparative bone. Similar to zebrafish, no differences in proliferation (pHH3 assay) or cell death (TUNEL) were detected in *Sox9-CreER;Smo*^*fl/fl*^ mice at early stages of bone repair, highlighting a distinct role of Hh signaling compared to that in previous reports of Hh signaling at the growth plate, where this pathway is required predominantly for chondrocyte proliferation rather than differentiation.^57^

### Hh signaling during appendage regeneration

Hh signaling is also important during appendage regeneration. Some urodeles can regenerate limbs and tails, while zebrafish can regenerate a variety of structures, including their fins, both via mechanisms that appear to be highly dependent on Hh signaling. Blocking Hh signaling with cyclopamine during limb regeneration in newts and tail regeneration in axolotls inhibited the proliferation of blastema cells and blocks regeneration.^72,73^ In addition, during axolotl tail regeneration, the induction of *Sox9*-expressing cartilage was inhibited. Cyclopamine treatment has also been reported to block mesenchymal proliferation and bone outgrowth during zebrafish fin regeneration.^74^ However, at least in zebrafish, the antiproliferative effect of cyclopamine during fin regeneration may be due to off-target activity. With another drug, BMS-833923, SHH-SMO signaling was inhibited with a higher specificity and was used to show that *shha* is required more for ray branching morphogenesis than for fin outgrowth. In *ihha*-deficient zebrafish, normal fin regeneration occurred, but defects in mineralization were observed. Interestingly, this phenomenon occurred without alterations in the activity of a *ptch2* reporter that is typically indicative of canonical Hh signaling. Therefore in zebrafish, *ihha* likely promotes mineralization in the regenerated fin through noncanonical Hh signaling. These results highlight the possible engagement of two types of Hh signaling in one injury context.

The regeneration observed in the species above likely reflects substantial dedifferentiation and redifferentiation (epimorphosis), strategies of repair that may not be as prevalent in mammals. Interestingly, regeneration in reptiles may involve some strategies employed during blastema-based regeneration and other strategies more similar to those in mammals. Lizards such as *Anolis carolinensis* will drop their tails (caudal autotomy) and then reform new tails that are functional but lack vertebrae extending to the tip. Some bone forms at the proximal cut end via a cartilage callus intermediate, similar to mammalian periosteal-mediated fracture repair. However, the distal portion regenerates a cartilage tube predominantly via blastema-based epimorphosis. This cartilage mineralizes but never ossifies. Treatment with cyclopamine during blastema stages (9 days post-amputation) blocked both the formation of a cartilage callus and a cartilage tube.^75^ When cyclopamine was applied later, to avoid affecting the blastema (28 or 42 days after amputation), endochondral ossification of the proximal cartilage callus and mineralization of the distal cartilage tube were inhibited.^76^ Thus, based on these results in lizard, Hh signaling may be required for blastema proliferation and differentiation during epimorphic regeneration and may promote ossification and cartilage differentiation, similar to mammalian fracture repair.

## Conclusion and unanswered questions

Despite conflicting reports and myriad models, stimulating the Hh signaling pathway appears to generally have a positive effect on bone repair; thus, the application of HH protein or pathway agonists remains a promising therapeutic strategy. However, to determine the best timing and delivery mechanism, additional in-depth analysis is still needed. At the onset of injury, it is still not known how cells become competent to respond to repair signals, which cells produce the HH ligand, or which HH ligand is required. Furthermore, the location of injury and the magnitude of injury may dictate the degree to which Hh signaling contributes to repair. At later repair stages, features of the injury location or injury type might determine whether stimulating the Hh pathway promotes differentiation of the cartilage callus or instead encourages bone formation.

Determining which cells respond to Hh signaling and which transcription factors are activated throughout the repair process will be important. As discussed earlier, important Hh pathway effectors include the GLI transcription factors GLI1, GLI2, and GLI3. GLI3 functions mainly as a repressor when Hh signaling is not active (GLI3-R), while GLI2 functions as the transcriptional activator when Hh signaling is active (GLI2-A). GLI1 appears to have redundant functions to GLI2 in osteogenesis and amplifies the response.^77–79^ During development, it has been shown that the actions of GLI2-A are not sufficient for normal bone development but also require the elimination of GLI3-R (^80^ and reviewed in ref. ^81^). In addition, the actions of GLI2-A and the elimination of GLI3-R can have different outcomes. For example, the absence of GLI3-R can restore the normal proliferation of chondrocytes in *Ihh* null mice but is not able to restore defects in osteoblast development.^82,83^ In the future, additional studies will hopefully determine the specific roles of these proteins in repair. For example, would stimulating GLI1-A or GLI2-A be sufficient to promote bone healing or would GLI3-R inactivation be required as well? Could GLI2-A play more of a role in bone formation while GLI3-R is important for cartilage or vice versa? Moreover, other signaling pathways can regulate GLI transcriptional activities during bone development, homeostasis and pathogenesis.^4,84^ Indeed, it is important to note that GLI activation may occur via noncanonical pathways that are not dependent on SMO or the presence of an HH ligand (Fig. 3). Furthermore, GLI-independent noncanonical Hh signaling may potentially be triggered by Hh ligands in addition to GLI activation upon injury. Additional studies, therefore, are needed to clarify the precise mechanisms that activate the canonical vs. noncanonical Hh pathway during bone repair in vivo. From a therapeutic standpoint, acquiring this in-depth understanding will be important not only to determine treatment timing but also to determine if Hh signaling has different roles in different repair contexts.

Outside of its roles within skeletal lineage cells, Hh signaling has additional roles in other cell types, such as osteoclasts, and the reestablishment of the vascular network during bone repair.^85,86^ During embryonic development, Hh signaling activates the expression of angiogenic growth factors, which leads to vascularization.^87^ In addition, the ability of Hh signaling to promote vascularization and branching has been observed in multiple studies using different cell types and culture conditions.^28,33,87^ For example, coculture studies where Hh promotes both angiogenesis and osteogenesis in vitro have been used as evidence to suggest that Hh signaling links angiogenesis and osteogenesis during bone repair.^88^ In vivo, stimulating Hh signaling during repair correlates with enhanced vasculogenesis, as showed by CD31 staining and the formation of a well-organized, more mature vascular network when compared to controls with, in some cases, double the vessel volume 14 days after injury compared to untreated controls.^28,33^ These results further demonstrate that stimulating the Hh pathway may not only have a positive impact on skeletal lineages but may also impact other cell types during the bone repair process.

It is also clear that gaining a better understanding of how bone repair occurs in general will be critical. Exciting new techniques and tools are emerging that will allow us to address many unanswered questions about bone repair generally and about the role of Hh signaling more specifically. Although the overall steps of bone repair have been well characterized, the signaling pathways, progenitor populations, and how they interact to facilitate repair are not completely understood.

Techniques such as single-cell RNA-seq (scRNA-seq)^89^ can be used to understand which types of cells contribute to the repair process and how their contribution might change over time or in the context of different types of injuries. In addition, scRNA-seq can be used to identify cell types that are responsive to drug treatments and genetic manipulations. For instance, it could be quite revealing to compare the impact of blocked or enhanced Hh signaling at various time points during repair to determine which cells are affected and at which step in their differentiation. In addition, this technique could help identify other important signaling pathways by examining how their components are increased or decreased in specific subpopulations. Additionally, scRNA-seq analysis can help determine the heterogeneity of the cell types involved and potentially identify new skeletal progenitor and niche subpopulations that were not known before.

One of the main limitations of progress in understanding skeletal biology is the availability of specific Cre lines to study bone repair. Although Cre lines are available that broadly mark periosteal, endosteal, and growth plate populations, we still do not know which subpopulations are critical for bone repair. Some of these Cre lines that have been used include *Sox9*,^44,90^ *Gli1*,^27^ *Axin2*,^91^ *αSMA*,^92^ *Cathepsin K*,^93^ *Gremlin1*,^94^ *LepR*,^95^ *Pax3*,^96^ and *MyoD*.^97^ While some of these genes mark interesting cell types, the hierarchy of these populations and how they interact or overlap with one another is still unclear. The development of new lines that allow Dre/Rox recombination that can be used in combination with Cre/Lox will certainly help.^98,99^ New subpopulations identified by scRNA-seq could lead to the development of new Cre or Dre lines that could be used to lineage trace or alter gene function.^100^ For further elucidation of the role of Hh signaling during repair, new conditional lines could be used to specifically target modulators in the Hh signaling cascade, such as HHIP (extracellular HH antagonist), GAS1, CDO, BOC (membrane-associated coreceptor modulators), and SUFU (intracellular inhibitor).^8^ Two recent studies used *Gli1-CreER* mice to track cells during injury. During both femoral fracture repair^27^ and cranial hole repair,^21^ Gli1^+^ lineage-traced cells contributed to tissue repair, and at least in the cranium, ablation of this population using a DTA strategy prevented full repair. An interesting future direction would be to determine if eliminating the SMO coreceptor from these cells would block their function. Furthermore, it would be interesting to determine whether this Gli1^+^ population shares any properties with the Sox9^+^ population that requires Hh signaling to mediate large-scale rib repair.^44^

Determining the relationship between different lineages will require new techniques to characterize the hierarchy of these populations. New barcoding techniques^101^ and pseudotime computational methods are being developed, which could be adapted to map lineage relationships.^102^ From these kinds of assays, one important outcome could be a more refined understanding of different bone repair mechanisms. Bone tissue may be repaired in more ways than previously imagined, and the mode used may depend on the injury type or location. Cranial defects and stress fractures appear to repair predominantly through direct ossification, while nonstabilized fractures heal through a combination of direct ossification and endochondral repair, with little known about what dictates these different modes. Additionally, there is still much to be learned about the role of dedifferentiation,^103,104^ transdifferentiation,^65,66^ osteochondral hybrid cells,^44,60^ and messenger/organizing cells^44,105^ in skeletal repair.

Although some studies show conflicting results, this may be due to incomplete analysis or a limited understanding of bone repair. Overall, a more in-depth understanding of how the bone repair process works will help us better understand the specific role of Hh signaling. Utilizing new techniques with Hh signaling in mind will help us determine when and where the pathway is required in specific bone injury contexts. The studies in this review have largely determined that Hh signaling has a positive effect on bone repair, and together, they indicate that Hh signaling may have multiple functions during repair. With more comprehensive knowledge about bone repair and Hh signaling, therapeutic strategies can be optimized for the treatment of different types of bone injuries in the future.
